# The Impact of Heat Islands on Mortality in Paris during the August 2003 Heat Wave

**DOI:** 10.1289/ehp.1103532

**Published:** 2011-09-01

**Authors:** Karine Laaidi, Abdelkrim Zeghnoun, Bénédicte Dousset, Philippe Bretin, Stéphanie Vandentorren, Emmanuel Giraudet, Pascal Beaudeau

**Affiliations:** 1 Institut de veille sanitaire, Département Santé Environnement, Saint-Maurice, France; 2 Hawaii Institute of Geophysics and Planetology, University of Hawaii, Honolulu, Hawaii, USA; 3 Géomer, UMR-6554 LETG (Littoral, Environnement, Télédétection, Géomatique) CNRS (Centre national de la recherche scientifique), Institut Universitaire Européen de la Mer, Plouzané, France

**Keywords:** climate change, heat wave, mortality, nighttime temperature, satellite imagery, urban heat islands, urban planning

## Abstract

Background: Heat waves have a drastic impact on urban populations, which could increase with climate change.

Objectives: We evaluated new indicators of elderly people’s exposure to heat in Paris, from a public health prevention perspective, using satellite thermal images.

Methods: We used a time series of 61 images from the satellites of the National Oceanic and Atmospheric Administration’s (NOAA) Advanced Very High Resolution Radiometer (AVHRR) taken from 1 to 13 August 2003 to produce thermal indicators of minimum, maximum, and mean surface temperatures and diurnal temperature amplitude, with different lags between the meteorological data and the health impact. Health data came from a case–control study involving 241 people ≥ 65 years of age who died in the city of Paris or the nearby suburban area of Val-de-Marne during the August 2003 heat wave, and 241 controls who were matched to cases on age, sex, and residential zone. For each person, we integrated the thermal indicators in a conditional logistic regression model, adjusted for age and other potential confounders. We computed odds ratios (ORs) comparing the 90th and 50th percentiles of the temperature differences between cases and controls for various indicators.

Results: Mortality risk was significantly associated with exposure for two indicators: minimum temperatures averaged for 1–13 August [for a 0.41°C increase, OR = 2.17; 95% confidence interval (CI): 1.14, 4.16] and minimum temperature averaged on the day of death and the 6 preceding days (for a 0.51°C increase: OR = 2.24; 95% CI: 1.03, 4.87).

Conclusions: Our results support the influence of night temperatures on the health impact of heat waves in urban areas. Urban heat exposure indicators based on satellite imagery have the potential to identify areas with higher risk of death, which could inform intervention decisions by key stakeholders.

In summer 2003, a major heat wave occurred in Europe, causing approximately 30,000 deaths, including nearly 15,000 in France (Fouillet et al. 2006; World Health Organization Europe 2003). According to the French national weather service (Météo-France), the summer mean temperature in 2003 was 3.7°C higher than the mean summer temperature for the period 1950–2006 (Bessemoulin et al. 2004). The likelihood of extreme high-temperature events could increase with climate change, with models projecting a mean rise in temperature ranging from 1.1°C to 6.4°C during the 21st century (Intergovernmental Panel on Climate Change 2007).

Heat waves are particularly intense in urban areas, where surface characteristics alter the partitioning of surface heat fluxes compared with nearby rural areas. This alteration is mainly due to *a*) replacement of vegetation by asphalt and concrete, which modifies the radiative energy fluxes and decreases the surface moisture available for evapotranspiration; *b*) changes in the near-surface air flow owing to the complicated geometry of streets and buildings; and *c*) production of anthropogenic heat. These changes generate a temperature difference between urban and rural areas, which is referred to as the “urban heat island effect.” At night, urban areas slowly release the heat absorbed during the day, which prevents the human body from recovering from daytime high-heat exposure. In a study in Chicago, Illinois, Peng et al. (2011) estimated that summer warming trends could significantly increase heat-related mortality, which is more likely in big cities where half the world population now lives (United Nations Environment Programme 2002).

In 2004, the French Institute for Public Health Surveillance (Institut de veille sanitaire; InVS) set up a watch/warning system for heat waves, in close collaboration with the meteorological services. The alerts are based on regional thresholds for minimum and maximum temperatures averaged over 3 days that correspond with a major impact on mortality (Pascal et al. 2006). In addition, the InVS conducted a case–control study to identify risk factors for elderly people living at home during the 2003 heat wave. Vandentorren et al. (2006) reported that lack of mobility was a major risk factor, followed by some preexisting medical conditions. Housing characteristics, such as a lack of thermal insulation and sleeping on the top floor directly below the roof, were associated with greater risk. Protective factors included dressing lightly and using cooling techniques and devices (Lorente et al. 2005; Vandentorren et al. 2006).

Vandentorren et al. (2006) also observed that the temperature around the building was a major risk factor. This temperature was estimated from a single Landsat satellite image acquired 9 August at 1017 hours Universal Time Coordinated (UTC). Indeed, urban temperatures are difficult to estimate from meteorological stations, which are often located in airports or parks [as is the case in Paris (Parc Montsouris)] and are not representative of the real living conditions of people downtown. Furthermore, the weather station network is too sparse to record the spatial variations of urban temperatures, which may pose an increased risk to human health. Those are best observed using satellite thermal infrared sensing.

The Landsat Thematic Mapper (Landsat-TM) image showed surface temperature differences up to 4°C within the city of Paris, which may result in a dramatic health impact according to an expected doubling of the risk of mortality per 1°C increase in temperature (Vandentorren et al. 2006).

In 2005, a large series of satellite infrared images, recorded over the Paris region during the August 2003 heat wave, was processed and analyzed to retrieve the diurnal variations of surface temperature (Dousset et al. 2007, 2011). That study was based on the simultaneous operation of three National Oceanic and Atmospheric Administration (NOAA) satellites providing up to six images per day, fully resolving the diurnal temperature cycle at 1-km spatial resolution. Results indicated large surface temperature gradients, contrasted daytime and nighttime warming patterns, a significant relationship between nighttime temperature and building density, and cooling effects from urban parks (Dousset et al. 2007, 2011).

Based on the joint analysis of the mortality and temperature data set, in the present study we aimed to better understand the health impact of heat waves in urban areas, assess the daily and cumulative minimum and maximum exposure to heat, and implement indicators of heat exposure for elderly people in relation to site of residence. Unlike in the previous InVS study (Vandentorren et al. 2006), we were able to take into account night and day temperatures and different lags between temperature exposure and death.

## Materials and Methods

*Study location and period.* The climate portion of our study covered the Paris metropolitan region, and the health portion included the city of Paris and the nearby suburban department of Val-de-Marne. These areas were selected because of their location in the regions most severely affected by the heat wave and because of their architectural variety (Vandentorren et al. 2006). The city of Paris has > 2.2 million inhabitants and a high population density (21,000/km^2^) and is characterized by compact urbanization. Conversely, the Val-de-Marne department has 1.3 million inhabitants and a low population density (5,300/km^2^) and is composed of high-rise buildings, residential detached houses, industrial buildings and warehouses, parks, and woods. This type of suburban area produces different heat-relevant characteristics than does the city of Paris.

We calculated climatic normals for 1974–2003 and mean temperatures for 2003 for the summer months of June, July, and August. The minimum and maximum air temperatures in the city of Paris and the Val-de-Marne department were obtained from the Paris automated weather stations in Montsouris Park and Saint-Maur, respectively.

The heat wave lasted 9 consecutive days, 4–13 August. Our previous study in 2003 concerned the highest mortality period between 8 and 13 August (Pirard et al. 2005). The short time period increases the probability that deaths are linked to heat. In the present study we analyzed the satellite data from 1 to 13 August, including 3 days before the heat wave. We did not consider the period after the heat wave because the possible mortality displacement was investigated previously by LeTertre et al. (2006), who found no harvesting effect of the heat wave until the beginning of September 2003.

*Remote sensing data.* The satellite data set consists of a time series of 61 thermal infrared images recorded during 1–13 August 2003 and a multispectral SPOT (Système Pour l’Observation de la Terre) High Resolution Visible (HRV) image of 13 July 2003. Images from the U.S. NOAA Advanced Very-High-Resolution Radiometer (AVHRR) were acquired by the high-resolution picture transmission receiving station at the Istituto Nazionale di Oceanographica et di Geofisica Sperimentale in Trieste, Italy. The images were selected according to small zenithal-viewing angles to ensure ground resolution close to 1 km and to minimize both atmospheric attenuation and directional effects. The images were geometrically corrected for earth rotation and curvature and then transformed into a map projection. The radiometer on board the NOAA polar-orbiting satellites scans in five spectral channels (from visible to thermal infrared); for each satellite pass, an image is produced for each channel. The surface albedo, cloudiness, and relative amount of vegetation (i.e., vegetation index) were computed. The cloudy pixels were flagged, and the land surface temperature (LST) was retrieved from the thermal infrared channel 4, as described by Dousset et al. (2011). This processing resulted in the production of 61 individual thermal images at 1-km resolution over the period of 1–13 August. We also constructed averaged LST images at six time intervals based on the NOAA satellite passes (i.e., in the hours between 0100 and 0300, 0400 and 0700, 0900 and 1200, 1200 and 1500, 1500 and 1800, and 2000 and 2300 UTC) during 4–13 August. Temperatures recorded by satellites and meteorological stations are intrinsically different. Temperatures recorded by satellites correspond to the radiant temperature of mostly horizontal surfaces such as streets and roofs included in a pixel, whereas those recorded by meteorological stations correspond to the ambient temperature 1.50 m above the surface at a given site.

Surface temperature is closely related to the surface characteristics and properties. To analyze these properties, we produced a land-cover classification from the 20-m spatial resolution SPOT image that included water, urban densely built areas, suburban residential areas, light bare soils, forests and woods, and lawns and fields.

*Health data.* In the present study, we used health data collected previously for a case–control study performed after the 2003 heat wave (Vandentorren et al. 2006). That study was performed to analyze differences in health and life conditions (age, sex, residence area, socioeconomic conditions, way of life, housing, health problems, behavioral adaptation to heat) among elderly people who died compared with those who shared characteristics of age, sex, and place of residence but lived. Information was collected by questionnaires in face-to-face settings when possible, or by telephone. All living participants (and relatives in the case of deceased persons) were informed about the objectives of the study and their right to access and correct the data concerning them, and gave written consent. The study was authorized by the Commission Nationale Informatique et Libertés, the national committee for information technology and liberties in France.

The target population was people ≥ 65 years of age who lived at home during the heat wave. The study population was limited to those living in Paris and the nearby department of Val-de-Marne, and the study aimed for an exhaustive sample of all the people ≥ 65 years of age who died during the heat wave (8–13 August 2003) (Vandentorren et al. 2006).

Cases were defined as people ≥ 65 years of age who died during the mortality peak (8–13 August) from all causes except accidental causes, suicide, or acute surgery complications (*n* = 241). All cases in the study had resided at their home in Paris or in the nearby department of Val-de-Marne for at least 24 hr before their hospitalization or death (if they died at home).

Controls were ≥ 65 years of age, lived in the study areas, and were at home during 8–13 August. These controls (*n* = 241) were matched with cases on age (within a maximum of 5 years, followed by adjustment on this same variable), sex, and residential zone [within one of five socioeconomically homogeneous zones defined by the average level of rents (in Paris) or the proportion living in low-income households (in Val-de-Marne)] (Institut d’Aménagement et d’Urbanisme d’Île-de-France/Institut national de la statistique et des études économiques 2000, 2001). Each homogeneous zone included approximately 24–150 pixels, so matching on residential zone was not equivalent to matching on exposure.

**Figure 1:**
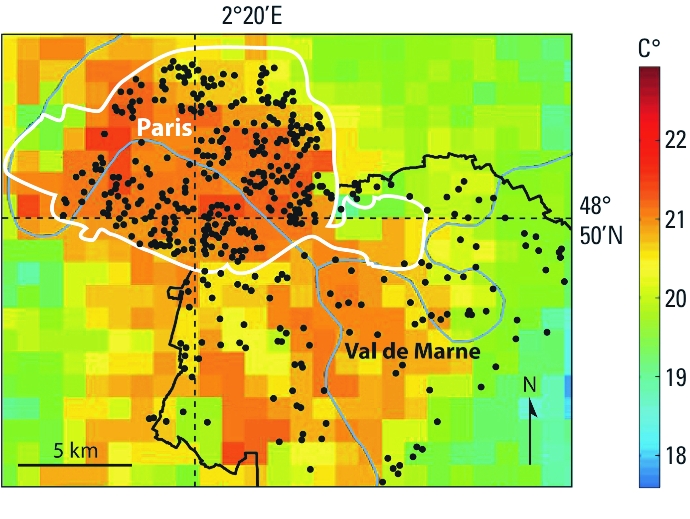
Spatial distribution of the geocoded addresses of cases and control in Paris and Val-de-Marne department (black dots) over the NOAA-AVHRR thermal image (channel 4) of 7 August 2003 at 0450 hours UTC. Reproduced from Dousset et al. (2011), with permission from John Wiley & Sons.

*Thermal indicators.* First, we geocoded the place of residence of each case and control. Then, in each of the 61 thermal images we extracted the temperature of the pixel that includes the geographic coordinates of each residence, which we considered the thermal indicator of the person at the time of measurement (Figure 1).

We constructed different indicators of temperature exposure based on daily minimum, maximum, and mean LST, as well as the temperature amplitudes (difference between maximum and minimum). We took into account different lags between temperature and death (1, 3, and 6 days), daily temperatures, and temperatures averaged over 3, 7, and 13 days. Lags were selected based on previous studies that suggested that heat waves have an immediate effect on mortality (heat stroke) occurring within 2 days (Kaiser et al. 2007), delayed effects on deaths occurring within 1 week, and delayed effects on respiratory diseases 7–14 days after the heat wave (Ballester et al. 1997).

*Data analysis.* The analysis was a case–control study matched on age, sex, and residential zone, based on a conditional logistic regression model (Breslow and Day 1980). We used the PHREG procedure of SAS/STAT software (release 8.02; SAS Institute Inc., Cary, NC, USA) to estimate odds ratios (ORs) associated with the different risk factors. We used the multivariate regression model developed in our previous study (Vandentorren et al. 2006). Specifically, relationships between the quantitative risk factors and mortality were initially studied nonparametrically to explore any possible nonlinear effects. Based on goodness of fit, we found that nonlinear transformation was not required. The multivariate analysis included the potential risk factors that were significantly associated with mortality (*p* < 0.05; age, occupation, social relations, behavioral adjustment to heat wave, mobility, history of disease, living conditions). The thermal indicator used to determine exposure in our previous study (Vandentorren et al. 2006) was based on a single image of 9 August from Landsat-TM satellite, which has a very high spatial resolution (120 m). However, its sensing time over Paris was not optimal to assess thermal patterns, and its 16-day repeat cycle precluded daily monitoring. We thus used the NOAA-AVHRR images to estimate a new thermal indicator for the present study. NOAA-AVHRR images have a lower spatial resolution (1 km) than do Landsat images, and they are collected twice a day. Furthermore, the availability of three satellites at the time of the heat wave resulted in an exceptional data set, increasing the available health data from 482 samples to > 28,000 samples from 1–13 August.

For each indicator, we determined the distribution of differences in the indicator between cases and controls and computed ORs for a temperature difference corresponding to the difference between the 90th and 50th percentiles of the distribution. For example, for the average mean temperature during 1–13 August, the median value (50th percentile) for the difference in mean temperature between cases and controls was 0.01°C and the 90th percentile of the difference was 0.38°C. Therefore, the OR for this indicator represents the increase in mortality associated with a 0.37°C increase in average mean temperature during 1–13 August.

## Results

The climate normals of Paris and the Val-de-Marne department (Table 1) show that average maximum temperatures were hotter at the Val-de-Marne station than at the Paris station, whereas average minimum temperatures were hotter at the Paris station than at the Val-de-Marne station in July and August but colder at the Paris station in June. Differences between stations were similar for summer 2003. These differences could be attributable to the situation of the Paris station in an urban park (Parc Montsouris).

**Table 1:** Mean air temperature (°C) from meteorological stations in Paris and Val-de-Marne.

Paris (Montsouris)					Val-de-Marne (Saint Maur)				
June	July	August	June	July	August
Mean minimum temperature												
1974–2003		13.55		15.56		15.62		14.08		14.76		14.62
2003		17.1		16.9		18.8		16.6		16.0		17.7
Mean maximum temperature												
1974–2003		22.56		24.56		25.22		25.41		26.03		26.58
2003		26.6		26.3		29.9		28.1		28.3		32.3

**Figure 2:**
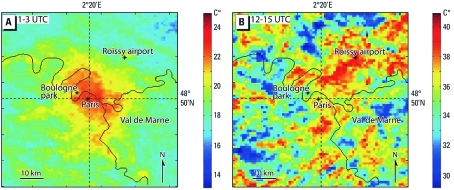
Median LST infrared images (channel 4) from 4–13 August 2003 averaged over 9 NOAA-AVHRR thermal images between 0100 and 0300 hours (1–3) UTC (*A*) and averaged over 10 NOAA-AVHRR thermal images between 1200 and 1500 hours (12–15) UTC (*B*). Reproduced from Dousset et al. (2011), with permission from John Wiley & Sons.

Figure 2 shows two averaged LST images of the Paris metropolitan area constructed from images collected between 0100 and 0300 hours UTC (9 images) and between 1200 and 1500 hours UTC (10 images) during 4–13 August. These images reveal spatial temperature gradients at the urban surface and contrasting nighttime and daytime heat island patterns, which reflect the different day and night rates of heating and cooling among urban, suburban, and rural areas. At night, a heat island was centered on downtown Paris, and during the day, multiple temperature anomalies were scattered in the densely built and industrial suburbs.

For all participants combined, recorded surface temperatures ranged from 12.2°C and 45.4°C (Table 2), with a median of 21.4°C at night (2000–0700 hours UTC) and 34.2°C during the day (0900–1800 hours UTC).

**Table 2:** LST distribution for 1–13 August 2003 in Paris and Val-de-Marne, derived from NOAA-AVHRR data.

No. of observations	Recorded LST (°C)								
	Minimum	25th percentile	50th percentile	75th percentile	90th percentile	95th percentile	Maximum	Mean ± SD
Total sample (cases plus controls)		28,838		12.2		21.3		25.9		33.9		36.8		38.1		45.4		27.3 ± 7.1
Controls		14,423		12.2		21.3		25.9		34.0		36.8		38.0		44.6		27.3 ± 7.1
Cases		14,415		12.2		21.3		25.9		34.0		36.8		38.2		45.4		27.3 ± 7.1
Difference in recorded surface temperatures between cases and controls (°C)		14,220		–6.09		–0.43		0		0.48		1.17		1.7		8.4		0.03 ± 1.03

**Figure 3:**
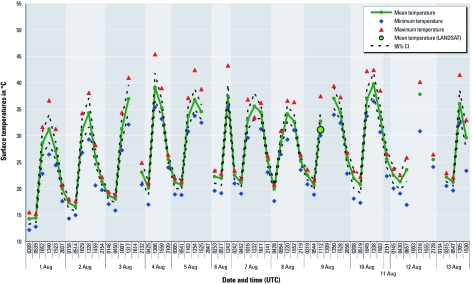
Diurnal cycle of LSTs at the addresses of the 482 cases and controls from 61 NOAA-AVHRR thermal images for 1–13 August 2003.

The diurnal cycle of the minimum, maximum, and mean surface temperatures observed at each time among the joined sets of all individuals (cases and controls) is plotted in Figure 3. Although Landsat and NOAA-AVHRR data are not quite comparable, the temperature of Landsat-TM for 9 August was 31°C at 1017 hours UTC (indicated by a green circle in Figure 3). Missing data correspond to images that were rejected because of cloudiness, noise within the image, or satellite-zenith angles above a given threshold. On average, the nighttime minimum temperatures were < 15°C on 1 August and then increased; the highest temperatures were recorded on 4, 7, 8, 9, 11, and 12 August. The highest daytime maximum temperatures also increased after 1 August and reached ≥ 40°C on 3, 4, 6, and 10 August.

We calculated the differences in recorded surface temperatures between matched cases and controls for 1–13 August for the hours when no data were missing (*n* = 14,220 cases and controls paired by day and time). These differences ranged from –6.09°C to 8.4°C (Table 2). The 90th percentile of these differences corresponds to 1.17°C, whereas the 50th percentile is equal to zero (i.e., no temperature difference between cases and controls).

The results of the conditional logistic regression show that two surface temperature indicators were significantly associated with the risk of death (*p* < 0.05): the average minimum temperature for 1–13 August [for a 0.41°C increase, OR = 2.17; 95% confidence interval (CI): 1.14, 4.16] and the average minimum temperature for the 6 days preceding death and the day of death (for a 0.51°C increase, OR = 2.24; 95% CI: 1.03, 4.87) (Table 3). The association between mortality and mean temperature for 1–13 August was marginally significant (*p* = 0.08), with an OR of 1.71 (for a 0.37°C increase: 95% CI: 0.94, 3.11). These ORs are consistent with the OR based on Landsat data for 9 August (for a 2.13°C increase: OR = 2.98; 95% CI: 1.37, 6.50) despite having a lower spatial resolution (Table 3).

**Table 3:** Results from the conditional logistic regression model for significant surface temperature indicators derived from NOAA-AVHRR data and the Landsat surface temperature indicator.

Satellite/thermal indicator	Difference in temperature (°C) between cases and controls*a*	OR (95% CI)*b*
Landsat				
Temperature on 9 August at 1017 hours UTC*		2.13		2.98 (1.37, 6.50)
NOAA				
1–13 August				
Average maximum temperature		0.97		1.29 (0.61, 2.72)
Average minimum temperature*		0.41		2.17 (1.14, 4.16)
Average mean temperature**		0.37		1.71 (0.94, 3.11)
From 2 days before death to the day of death				
Average maximum temperature		1.25		0.91 (0.56, 1.47)
Average minimum temperature		0.68		1.40 (0.67, 2.91)
Average mean temperature		0.67		1.06 (0.60, 1.89)
From 6 days before death to the day of death				
Average maximum temperature		0.92		0.93 (0.51, 1.69)
Average minimum temperature*		0.51		2.24 (1.03, 4.87)
Average mean temperature		0.46		1.34 (0.71, 2.51)
The day before death				
Maximum temperature		1.51		1.05 (0.77, 1.42)
Minimum temperature		0.85		1.17 (0.70, 1.96)
Mean temperature		0.86		1.37 (0.86, 2.20)
The day of death				
Maximum temperature		1.62		0.87 (0.63, 1.22)
Minimum temperature		0.64		0.88 (0.52, 1.46)
Mean temperature		0.77		0.75 (0.52, 1.09)
Mean temperature amplitude				
1–13 August		0.91		0.83 (0.42, 1.62)
From 2 days before death to the day of death		1.25		0.84 (0.52, 1.36)
From 6 days before death to the day of death		0.96		0.70 (0.38, 1.29)
The day before death		1.72		1.02 (0.71, 1.45)
The day of death		1.80		0.88 (0.61, 1.27)
**a**90th percentile – 50th percentile. **b**Computed using the 90th and 50th percentiles of the temperature differences between cases and controls. *Significant (*p* < 0.05). **Almost significant (*p* = 0.08).				

## Discussion

This study demonstrated that, in elderly individuals, exposure to a high nighttime temperature over several days increases the probability of death during a heat wave in urban conditions, whereas daytime temperature is less important (and in this study not significant). This combination of hot days and nights weakens the body, which cannot recover, and can lead to death (Besancenot 2002).

Potential bias linked to the health data were described previously by Vandentorren et al. (2006). The bias is related to case definition (which was not very specific), the selection of the controls (people who agreed to act as controls were well enough to answer our questions, so the control group may have overrepresented people in good health), and information bias (the case or control status was known by the investigator and the controls). To better characterize this bias, we could have investigated the changes that would result from using the addresses of the potential controls who did not participate in this study, but we did not have the detailed information (e.g., socioeconomic, pathologies, drugs, housing) on these people necessary to implement the model. However, we matched on age, sex, and residential zone to reduce confounding bias.

In addition, given the 1-km spatial resolution of NOAA images, some case–control pairs were located in the same pixel, which so corresponded to the same surface temperature. Thus, spatial matching had the unfortunate effect of minimizing exposure differences, reducing study power. Indeed, we found higher temperature differences between cases and controls for the Landsat indicator because of its higher spatial resolution, and lower differences for the NOAA indicators because temperatures were smoothed over wider pixels. However, we found significant ORs, particularly for nighttime surface temperature indicators. We can assume that ORs would have been higher with better spatial resolution because address misclassification would have been reduced, especially for nighttime temperature, which was more strongly associated with mortality than was daytime temperature. In this study, we observed that elderly people who were most exposed to high surface temperatures, especially during the night, had a higher risk (more than double, ORs ~ 2) of dying during the August 2003 heat wave than those who where less exposed (as in most case–control studies, the OR is a good estimation of the relative risk).

Comparing the Landsat-TM to the NOAA-AVHRR temperature indicators, we observed that the loss in spatial resolution with NOAA-AVHRR is counterbalanced by the gain in image periodicity and the availability of nighttime temperatures. Smargiassi et al. (2009) found similar results concerning the impact of surface temperatures in Montreal, Ontario, Canada. They compared daily mortality data from 1990 to 2003 to the surface temperature data from two Landsat-TM satellite images (1990 and 2001) using a case-crossover design. As in our study, they observed that the risk of death was higher in hot spots. In addition, they found that risk did not differ for nonaccidental causes of death or for cardiovascular deaths but were higher for respiratory deaths. However, the use of only two satellite thermal images over a 14-year period precludes any climate and health statistical study.

The impact of heat in urban areas will increase along with climate change. For example, in a Chinese study, Tan et al. (2010) reported that from 1975 to 2004, the number of hot days and heat-related mortality increased more rapidly in urban areas than in rural areas. This highlights the significance of our study and the potential applications to urban strategies and policies of mitigation and adaption in the context of a summer warming trend.

Experiences in several countries have shown that heat-related deaths are largely preventable through appropriate communication and prevention, mainly via heat prevention plans (Fouillet et al. 2008; Kovats and Hajat 2008; Naughton et al. 2002). Specifically, for long-term prevention in the case of urban heat islands, a range of protective measures could be planned in areas identified as being hotter at night. Well-targeted protective measures imply accurate knowledge of surface properties and physical processes that generate urban heat islands. The latter result in part from a decrease in evaporation, anthropogenic heat emission, and heat retention within buildings; these parameters can be improved. For example, urban parks have significant cooling effects. Previous research in the Paris region (Dousset and Gourmelon 2003) indicated a strong negative correlation between summer afternoon temperatures and vegetation index and a 0.2°C decrease per unit of vegetation index during the heat wave (Dousset et al. 2011). Indeed, the evapotranspiration from vegetation decreases the sensible heat, hence the temperature. Shadowed surfaces can be cooler by 11–25°C compared with those exposed to sun (Akbari et al. 1997), and evapotranspiration, alone or associated with shadow, can reduce the summer temperature peak by 1–5°C (Huang et al. 1990; Kurn et al. 1994). Vegetated roofs (sometimes known as “green roofs”) have good thermal performance, with surface temperatures similar to or slightly cooler than air temperatures, whereas conventional roofs can reach up to 50°C (Liu and Baskaran 2003). Roofs covered with selected white paints or highly reflective materials (sometimes known as “cool roofs”) reduce heat in the buildings below. During a heat wave, such properties enable these roofs to cool off by 28–33°C [U.S. Environmental Protection Agency (EPA) 2011].

When air temperature is > 35°C, vegetation can also have an impact on ozone production because it may release more isoprene, which acts as a catalyst (Lewis et al. 2000). Despite this, many studies have shown the positive impact of vegetation, which reduces both the urban heat island and air pollution, owing to dry deposition properties. Tree planting and green roofs both reduce pollution, with the greatest reduction for ozone, followed by nitrogen dioxide, particulate matter < 10 µm in aerodynamic diameter, and sulfur dioxide (McDonald et al. 2007; Nowak et al. 2000; Yang et al. 2008). Because the impact of high temperatures on mortality is far more important than the impact of ozone during a heat wave (Filleul et al. 2006), planting trees and green roofs could be an effective option to mitigate urban heat islands. Cool pavements, created using emerging technologies, are also being tested in some cities; for example, light-colored porous concrete allows water infiltration, enhances water evaporation, and partly reflects solar radiation. Given the percentage of impervious pavement in cities, it is an important element to consider in heat island mitigation (U.S. EPA 2011).

The link between indoor and outdoor temperature should also be taken into account to adapt heat reduction strategies in cities. Thus, a study conducted in Montreal in July 2005 on 75 apartments (Smargiassi et al. 2008) showed linear relations between outdoor air, surface temperature, and indoor temperature. The indoor temperature was higher in big buildings, which should be the first target for preventive measures. The surface temperature of the preceding 24 or 72 hr also affects the indoor temperature (Smargiassi et al. 2008; Wright et al. 2005).

## Conclusion

This joint study has allowed us to estimate the impact of heat exposure on an elderly population in urban locations, and our findings highlight the role of high nighttime temperatures and the duration of heat. Our findings are relevant to long-term prevention in the framework of national and local heat wave plans and should be of interest to urban decision makers, including health and environment ministries, mayors, and urban planning agencies, to guide actions for reducing heat islands.

Because we spend a great part of our time inside buildings (at home or at work), further studies are needed to better understand the relationship between outdoor and indoor temperatures. In the case of an extreme heat event, the elderly are the most at risk because they sometimes spend all day inside poorly ventilated homes or uninsulated apartments on top floors of buildings (Zeghnoun and Dor 2010).
